# Knowledge-Aided Structured Covariance Matrix Estimator Applied for Radar Sensor Signal Detection

**DOI:** 10.3390/s19030664

**Published:** 2019-02-06

**Authors:** Naixin Kang, Zheran Shang, Qinglei Du

**Affiliations:** 1 Unit 93046 of PLA, Qingdao 266111, China; kangkangnaixin@163.com; 2 School of Electronic Science, National University of Defense Technology, Changsha 410073, China; 3 Air Force Early Warning Academy, Wuhan 430019, China; dql822@163.com

**Keywords:** covariance estimation, knowledge-aided, radar sensor, signal detection

## Abstract

This study deals with the problem of covariance matrix estimation for radar sensor signal detection applications with insufficient secondary data in non-Gaussian clutter. According to the Euclidean mean, the authors combined an available prior covariance matrix with the persymmetric structure covariance estimator, symmetric structure covariance estimator, and Toeplitz structure covariance estimator, respectively, to derive three knowledge-aided structured covariance estimators. At the analysis stage, the authors assess the performance of the proposed estimators in estimation accuracy and detection probability. The analysis is conducted both on the simulated data and real sea clutter data collected by the IPIX radar sensor system. The results show that the knowledge-aided Toeplitz structure covariance estimator (KA-T) has the best performance both in estimation and detection, and the knowledge-aided persymmetric structure covariance estimator (KA-P) has similar performance with the knowledge-aided symmetric structure covariance estimator (KA-S). Moreover, compared with existing knowledge-aided estimator, the proposed estimators can obtain better performance when secondary data are insufficient.

## 1. Introduction

Covariance matrix estimation plays an important role in advanced radar sensor signal processing algorithms such as space–time adaptive processing (STAP) [1] and adaptive signal detection [2,3,4,5,6]. In the homogeneous Gaussian environment, the sample covariance matrix (SCM) estimator [7] is the maximum likelihood estimate (MLE) [8]. However, in many situations, the clutter is not Gaussian distributed. In K-distributed clutter, a special case of compound-Gaussian distributed clutter, the normalized SCM (NSCM) estimator [9] is often used to estimate the actual covariance matrix. In correlated heavy-tailed clutter, the approximate maximum likelihood (AML) estimator [10,11] was derived, which is an iterative solution of covariance matrix, as well as a unified method of the SCM and NSCM estimator. In a partially homogeneous clutter environment, an iterative method was provided to calculate the weighting coefficients of each covariance matrix of secondary data vector [12].

Recently, a covariance estimate based on the geometric means and medians was proposed in the open literature. In [13,14], the authors used five difference geometric means (Euclidean mean, Log–Euclidean mean, Root–Euclidean mean, Power-Euclidean mean, and Cholesky mean) to obtain the corresponding covariance estimators without considering the clutter distribution. In [15], the authors used the geometric means and medians to obtain the estimators and obtained the corresponding detectors. Using geometric methods can obtain the non-linear model between primary data covariance matrix and secondary data covariance matrix which can further improve the covariance estimation accuracy. However, in the real world, above methods will suffer from the sample-starvation effect, namely, we may not obtain sufficient secondary data samples to estimate an accurate covariance matrix. Precisely, to ensure the loss within 3 dB, the required number of secondary data are near twice the number of the system degrees of freedom (DOF), according to the Reed, Mallett, and Brennan (RMB) [16] criterion.

In order to alleviate the sample-starvation effect, a priori knowledge of the clutter covariance matrix is a possible way to improve covariance matrix estimation accuracy [17]. The knowledge-aided (KA) technology, as an important part of a cognitive radar system [18], has been widely used in the secondary data selection [19], detector design [20,21,22], and covariance matrix estimation [23,24,25]. The essence of KA method is to use the prior information to reduce the DOF of covariance matrix estimation, and hence obtain a more accurate result in small sample case [26].

The KA covariance estimation methods can fall loosely into three categories based on the difference of knowledge. The first category of knowledge is the structure information of covariance matrices, such as symmetric spectrum [21,27], persymmetry structure [20,22,28] and identity matrix [29,30,31]. The second category of knowledge is the prior statistical distribution knowledge of the data of covariance matrix, such as the Wishart or inverse Wishart distributed [32,33,34]. The third category of knowledge is the data of environment, such as synthetic aperture radar (SAR) imagery [35], physics-based models [36], digital elevation model (DEM) [37], and history or simulation data of clutter [38,39].

The structure of the covariance matrix can reduce the covariance matrix degree of freedom, thereby reducing the dependence on the number of samples. Data of the environment can be used to obtain an a priori clutter covariance matrix and utilize this covariance matrix combined with the SCM estimator to obtain a more accurate one, which is usually in the form of color-loaded version. The color-loaded methods can result in the linear combination of the prior covariance matrix and the SCM estimator. The key issue of these methods is to determine the weighting factor. In the Gaussian environment, Ref. [23] used the convex combination (CC) of the prior covariance estimator and SCM estimator and obtained the weighting factor via solving a convex optimization problem. In [24], the authors used a maximum likelihood (ML) estimator approach to obtain the optimal weighting factor via a search method in the Gaussian environment. These methods are based on a Gaussian distribution to obtain the weighting factor but the real world is not always Gaussian. Therefore, existing KA methods can solve the sample-starvation problem but they will suffer from loss due to model mismatch when the clutter is non-Gaussian [40,41,42].

In addition, another class of color-loaded methods use the Bayes framework to obtain covariance matrix estimation. These methods [43,44,45] assumed the covariance matrix follows some prior distributions such as Winshart [17] and inverse-Winshart [34] distribution and use the prior covariance matrix as the mean value of the distributions to caculate the maximum posterior probability (MAP) estimation of the covariance.

In this paper, we use the linear combination of the prior covariance matrix and the estimated covariance matrix to obtain the knowledge-aided covariance estimators. It is different with the Bayes knowledge-aided covariance estimators which need assume the prior distribution of covariance. The main contribution in this work is a new method to calculate the weighting factor and combining the prior covariance with the structured covariance estimator via the new method to obtain three knowledge-aided structured covariance estimators. We show that the proposed estimators can use knowledge effectively and have a better estimation and detection performance both in Gaussian and non-Gaussian clutter when secondary data are insufficient. These results are valiadted by numerical experiments and real sea clutter data collected by the IPIX radar sensor system.

The rest of this paper is organized as follows. Section 2 gives the data model and a brief description of the Euclidean mean. The proposed estimators in this paper are detailed in Section 3. Results obtained from experiments are presented in Section 4. Section 5 concludes the paper.

## 2. Problem Formulation

In this section, we derive some classic covariance estimators from the view of the Euclidean metric. First, we formulate the signal model. Then, we obtain the classic covariance estimators via Euclidean mean.

### 2.1. Data Model

Consider a set of received radar sensor data where the cell under test (CUT) and secondary data share the same covariance structure. Specifically, denoted by $r_{0}$, $r_{k}$, $\left(k=1,\cdots ,K\right)$, the N−dimensional random vectors. Assuming $r_{0}$ and $r_{k}$ are complex circular multivariate compound-Gaussian distributed, which can be expressed as [46,47]

(1) $$\left\{\begin{array}{c} r_{0}=\sqrt{\tau_{0}}g\\ r_{k}=\sqrt{\tau_{k}}g\begin{array}{cc} , & \left(k=1,2,\ldots ,K\right) \end{array} \end{array}\right.$$

where $\sqrt{\tau_{0}}$ and $\sqrt{\tau_{k}}$ are positive and possibly random numbers, named the texture component, determing the local scattering power, and g is modeled as N−dimensional circularly symmetric zero-mean vectors with positive definite covariance matrix

(2) $$E\left[gg^{H}\right]=\Sigma$$

where ${\left(\cdot \right)}^{H}$ denotes the conjugate transpose operator and E(·) denotes the statistical expectation. Therefore, the conditional covariance matrix of $r_{0}$ and $r_{k}$ for a given $\tau {}_{0}$, $\tau {}_{k}$ are $R_{0}=E\left(r_{0}{r_{0}}^{H}|\tau {}_{0}\right)=\tau_{0}\Sigma$ and $R_{k}=E\left(r_{k}{r_{k}}^{H}|\tau {}_{k}\right)=\tau_{k}\Sigma$, respectively.

### 2.2. Euclidean Mean

From the geometry metric perspective, the Frobenius normal of two matrices is the Euclidean distance between them, namely

(3) $${∥A-B∥}_{F}=\sqrt{\mathrm{trace}\left[\left(A-B\right){\left(A-B\right)}^{H}\right]}=d_{E}\left(A,B\right)$$

where $d_{E}\left(\cdot ,\cdot \right)$ denotes the Euclidean distance.

In number field, for a set of *K* positive numbers $\left\{x_{1},x_{2},\cdots ,x_{K}\right\}$, the mean $\bar{x}$ is the minimum value of the sum of the squared distance to the given points

(4) $$\bar{x}=\underset{x>0}{\arg\min}\;\frac{1}{K}\sum_{i=1}^{K}{\left|x-x_{i}\right|}^{2}$$

In the geometry field, the mean for a finite set of Hermitian positive definite (HPD) matrices origins from generalized mean for positive numbers. Pennec [48] defined the geometry means, and obtained the geometric mean using a gradient descent algorithm. Pennec proved that the geometry means for a set of HPD matrices exist and unique. Therefore, the Euclidean mean associated with Euclidean distance (3), of a set of *K* HPD matrices $\left\{{\hat{R}}_{1},{\hat{R}}_{2},\cdots ,{\hat{R}}_{K}\right\}$, is defined by

(5) $$\bar{R}=\underset{R≻0}{\arg\min}\;\frac{1}{K}\sum_{k=1}^{K}d_{E}^{2}\left(R,{\hat{R}}_{k}\right)$$

Then, the Euclidean mean can be derived by differentiating R

(6) $$\hat{R}=\frac{1}{K}\sum_{k=1}^{K}{\hat{R}}_{k}$$

It is noted that Equation (6) is the SCM estimator or the NSCM estimator when ${\hat{R}}_{k}=r_{k}r_{k}^{H}$ or ${\hat{R}}_{k}=\frac{Nr_{k}r_{k}^{H}}{r_{k}^{H}r_{k}}$, respectively. The SCM estimator and the NSCM estimator are obtained based on Gaussian clutter and non-Gaussian clutter, respectively. Therefore, in the Euclidean metric case, covariance estimate methods based on probability distributed are equivalent to the geometric metric methods. Moreover, to eliminate the effect of texture components $\tau_{0}$ and $\tau_{k}$, like the NSCM, we normalized the received data vectors, namely,

(7) $$z_{k}=\frac{r_{k}}{\sqrt{r_{k}^{H}r_{k}/N\;}},k=0,1,\cdots ,K$$

where $r_{k}^{H}r_{k}/N$ is the moment estimation of texture component [9,11].

In the Gaussian clutter, $\tau {}_{0}$ and $\tau {}_{k}$ are constant and equal to each other, the SCM estimator is the maximum estimation (ML) estimator of the CUT. In the compound-Gaussian clutter, $\tau {}_{0}$ and $\tau {}_{k}$ are positive random variables and usually unequal, and the SCM estimator suffers from the serious performance loss. In contrast, the NSCM can perform well in such an environment. However, to ensure the loss within 3 dB, both of these estimators should follow the RMB criterion. Unfortunately, in the real world, the secondary data are always insufficient which reduces the estimation performance of the SCM and NSCM estimators. Using prior knowledge can solve this problem effectively. In the next section, we provide a new knowledge-aided covariance estimate method via Euclidean metric.

## 3. Proposed Estimators

In this section, we derive our covariance estimator. We use the linear combination as the covariance estimation model. The covariance estimation model of each cell is modeled as [23]

(8) $$\begin{array}{cc} {\hat{R}}_{k0}=\alpha R_{A}+\left(1-\alpha \right){\hat{R}}_{k}, & 0\leq \alpha \leq 1 \end{array}$$

where $R_{A}$ is the prior covariance matrix of CUT, which can be obtained from the SAR imagery [35], physics-based models [36], digital elevation model (DEM) [37] and history or simulation data [38,39], and α is the weighting factor. Then substituting Equation (8) into Equation (5)

(9) $$\begin{array}{cc} \bar{R} & =\underset{R≻0}{\arg\min}\;\frac{1}{K}\sum_{k=1}^{K}d_{E}^{2}\left(R,{\hat{R}}_{k0}\right)\\ & =\underset{R≻0}{\arg\min}\;\frac{1}{K}\sum_{k=1}^{K}{∥R-\alpha R_{A}-\left(1-\alpha \right){\hat{R}}_{k}∥}_{F}^{2} \end{array}$$

First, taking the derivative of Equation (9) with respect to R results in

(10) $${\hat{R}}_{\mathrm{KA}}=\frac{1}{K}\sum_{i=1}^{K}{\hat{R}}_{k0}=\frac{1}{K}\sum_{i=1}^{K}\left(\alpha R_{A}+\left(1-\alpha \right){\hat{R}}_{k}\right)$$

Second, taking the derivative of Equation (9) with respect to α and nulling the result, we obtain (See Appendix A for a detailed derivation)

(11) $$\hat{\alpha }=\frac{\sum_{k=1}^{K}{∥{\hat{R}}_{k}-\hat{R}∥}_{F}^{2}}{\sum_{k=1}^{K}{∥R_{A}-{\hat{R}}_{k}∥}_{F}^{2}}$$

where $\hat{R}$ is the Euclidean mean of R.

It is noted that when ${\hat{R}}_{k}=r_{k}r_{k}^{H}$ and $\hat{R}={\hat{R}}_{\mathrm{SCM}}$ we can rewrite $\hat{\alpha }$ as

(12) $$\begin{array}{cc} \hat{\alpha } & =\frac{\frac{1}{K^{2}}\sum_{k=1}^{K}{∥{\hat{R}}_{k}-{\hat{R}}_{\mathrm{SCM}}∥}_{F}^{2}}{\frac{1}{K^{2}}\sum_{k=1}^{K}{∥R_{A}-{\hat{R}}_{k}∥}_{F}^{2}}\\ & =\frac{\frac{1}{K^{2}}\sum_{k=1}^{K}{∥r_{k}r_{k}^{H}-{\hat{R}}_{\mathrm{SCM}}∥}_{F}^{2}}{{∥\frac{1}{K}\sum_{k=1}^{K}R_{A}-\frac{1}{K}\sum_{k=1}^{K}{\hat{R}}_{k}∥}_{F}^{2}}\\ & =\frac{\rho }{{∥R_{A}-\hat{R}∥}_{F}} \end{array}$$

where $\rho =\frac{1}{K^{2}}\sum_{k=1}^{K}{∥x_{k}x_{k}^{H}-{\hat{R}}_{\mathrm{SCM}}∥}_{F}^{2}$. And Equation (12) is similar with the Equation (20) in [23].

In this study, we use structure information to improve the estimation accuracy of ${\hat{R}}_{k}$ and $\hat{R}$. This structure information can be obtained via prior knowledge.

- *Persymmetric covariance matrix*
  For a radar sensor system using a symmetrically spaced linear array with constant pulse repetition interval, the covariance matrix has the persymmetric structure [49]. This structure information yields the estimate [28]
  (13) $${\hat{R}}_{kP}=\frac{1}{2}\left[z_{k}z_{k}^{H}+J{\left(z_{k}z_{k}^{H}\right)}^{*}J\right]$$
  and
  (14) $${\hat{R}}_{P}=\frac{1}{2K}\sum_{k=1}^{K}\left[z_{k}z_{k}^{H}+J{\left(z_{k}z_{k}^{H}\right)}^{*}J\right]$$
  where ${\left\{\cdot \right\}}^{*}$ denotes complex conjugate, and J is the permutation matrix, i.e.,
  (15) $$J=\left[\begin{array}{ccccc} 0 & 0 & \cdots & 0 & 1\\ 0 & 0 & \ldots & 1 & 0\\ \vdots & \vdots & \vdots & \vdots & \vdots \\ 1 & 0 & 0 & 0 & 0 \end{array}\right]$$
- *Symmetric covariance matrix*
  For a temporal steering and fixed radar sensor system, the symmetric property of the clutter power spectral density (PSD) is always present [21]. This structure information yields the estimate
  (16) $${\hat{R}}_{kS}=ℜ\left\{z_{k}z_{k}^{H}\right\}$$
  and
  (17) $${\hat{R}}_{S}=\frac{1}{K}\sum_{k=1}^{K}ℜ\left\{z_{k}z_{k}^{H}\right\}$$
  where $ℜ\left\{\cdot \right\}$ denotes the real part of $z_{k}z_{k}^{H}$.
- *Toeplitz covariance matrix*
  If the data obtained by sampling a spatially stationary noise field with a uniform linear array, then the Toeplitz structure exists [50]. The Toeplitz structure covariance matrix can be calculated as [15]
  (18) $$\begin{array}{c} R_{kT}=E\left[z_{k}z_{k}^{H}\right]=\left[\begin{array}{cccc} t_{0} & t_{1}^{*} & \cdots & t_{N-1}^{*}\\ t_{1} & t_{0} & \cdots & t_{N-2}^{*}\\ \vdots & \ddots & \ddots & \vdots \\ t_{N-1} & \cdots & t_{1} & t_{0} \end{array}\right]\\ \begin{array}{cc} t_{j}=E\left[z_{i}z_{i+j}^{*}\right], & \begin{array}{cc} 0\leq j\leq N-1, & 1\leq i\leq N \end{array} \end{array} \end{array}$$
  where $t_{j}=E\left[z_{i}z_{i+j}^{*}\right]$ is the correlation coefficient. $R_{kT}$ is a Toeplitz matrix with $R_{kT}^{H}=R_{kT}$. According to the ergodicity of a wide-sense stationary, the correlation coefficient of data $r_{k}$ can be calculated by averaging over time instead of its statistical expectation, as
  (19) $$\begin{array}{cc} {\hat{t}}_{j}=\frac{1}{N-j}\sum_{n=1}^{N-1-j}z_{k}\left(n\right)z_{k}^{*}\left(n+j\right), & 0\leq j\leq N-1 \end{array}$$
  where $z_{k}\left(n\right)$ is the *n*th element of $z_{k}$,
  (20) $${\hat{R}}_{T}=\frac{1}{K}\sum_{k=1}^{K}{\hat{R}}_{kT}$$

Hence, we obtain three knowledge-aided estimators via three structure information, namely, the knowledge-aided persymmetric covariance estimator (KA-P), the knowledge-aided symmetric covariance estimator (KA-S) and the knowledge-aided Toeplitz covariance estimator (KA-T). The detailed calculation method is shown in Table 1.

## 4. Performance Assessment

In this section, we analyze the estimation and detection performance of the proposed estimators with the existing SCM [7], CC [23] and ML [24] estimators. First, we compare estimation performance of the proposed estimators with the existing estimators. Second, we use the adaptive normalized matched filter (ANMF) detector [51,52] with each estimator to further illustrate the detection performance of the proposed estimators. All of these performance analyses are simulated in Gaussian and compound-Gaussian clutter. Finally, we analyze the detection performance via real sea clutter data collected by the IPIX radar sensor system. In all simulations, we consider a coherent pulse train of N=8 for reducing computation burden.

The prior covariance matrix $R_{A}$ is taken as a perturbed matrix of the actual matrix R and can be generated by [24]

(21) $$R_{A}=R⊙tt^{H}$$

where ⊙ denotes the Hadamard matrix product, and $t=\left[t_{1},t_{2},\cdots ,t_{N}\right]$ is a vector of independent and identically distributed (IID) Gaussian random variables with mean 1 and variance $\sigma^{2}$ $\left(t_{i}∼N\left(1,\sigma^{2}\right),\right.$ $\left.i=1,2,\cdots ,N\right)$. The perturbed vector t determines the effectiveness of the prior covariance matrix $R_{A}$, when the variance $\sigma^{2}$ is large ($\sigma^{2}>0.5$), the $R_{A}$ can be regarded as an invalid prior covariance matrix and the weighting factor α will become small, while the variance $\sigma^{2}$ is small the $R_{A}$ can be regarded as an effective prior covariance matrix and the weighting factor α will become large.

The one-lag correlation coefficient ρ=0.95 and normalized doppler of clutter $f_{c}=0.2$ are used to simulate the covariance matrix of clutter

(22) $$R_{k}\left(i,j\right)=\tau_{k}\rho^{\left|i-j\right|}\exp\left(\left(i-j\right)f_{c}\right),1\leq i,j\leq N,0\leq k\leq K$$

The texture component of compound-Gaussian clutter in each cell, namely $\tau_{k}$, follows the inverse gamma distribution [47,53] with shape parameter λ=3 and scale parameter μ=1, i.e.,

(23) $$f\left(\tau_{k}\right)=\frac{1}{\mu^{\lambda }\Gamma \left(\lambda \right)}\tau_{k}^{-(\lambda +1)}\exp\left(-\frac{1}{\mu \tau_{k}}\right)$$

### 4.1. Estimation Performance

In this section, we use the simulation data to measure the estimation performance of our estimators. First, we compare the estimation value of weighting factor α under difference secondary data and clutter environment. Second, we calculate the normalized Frobenius norm (NFN) of the error matrix [11] to display the estimation accuracy of the estimators.

**Figure 1:**
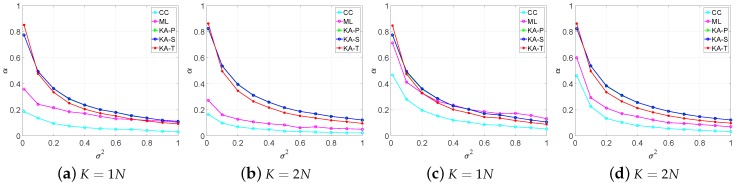
Estimation values of α under different values of the perturbation level clutter, (**a**,**b**) in compound-Gaussian clutter; (**c**,**d**) in Gaussian clutter.

Figure 1 illustrates that for the proposed estimators, the clutter environment and the number of secondary data have a little impact on the weighting factor. The proposed estimators are more sensitive to $\sigma^{2}$. When $\sigma^{2}$ becomes large α becomes small to keep the estimation accuracy. Therefore, the proposed estimators have the robust estimate property. The change in α of the KA-P and KA-S estimators show that these two estimators have similar performance in estimation. For the CC and ML estimators, the clutter environment has a significant impact on the weighting factor. Especially, in the compound-Gaussian clutter (Figure 1a,b), though the prior covariance matrix is accurate the weighting factor of the CC and ML is small. Therefore, the CC and ML estimators can not make the most of the prior knowledge under the compound-Gaussian clutter. Moreover, as the $\sigma^{2}$ increases, all the estimators tend to abandon the inaccurate prior knowledge. Therefore, all the KA methods can recognize the invalid knowledge.

Next, we measure the estimation accuracy by calculating the NFN of the error matrix, defined as [11]

(24) $${\mathrm{Error}}_{\mathrm{NFN}}≜\frac{E\left\{{∥\hat{R}-R∥}_{F}\right\}}{{∥R∥}_{F}}$$

where $\hat{R}$ can be replaced by ${\hat{R}}_{\mathrm{KA}-P}$, ${\hat{R}}_{\mathrm{KA}-S}$, ${\hat{R}}_{\mathrm{KA}-T}$, ${\hat{R}}_{\mathrm{SCM}}$, ${\hat{R}}_{\mathrm{CC}}$ [23] and ${\hat{R}}_{\mathrm{ML}}$ [24].

**Figure 2:**
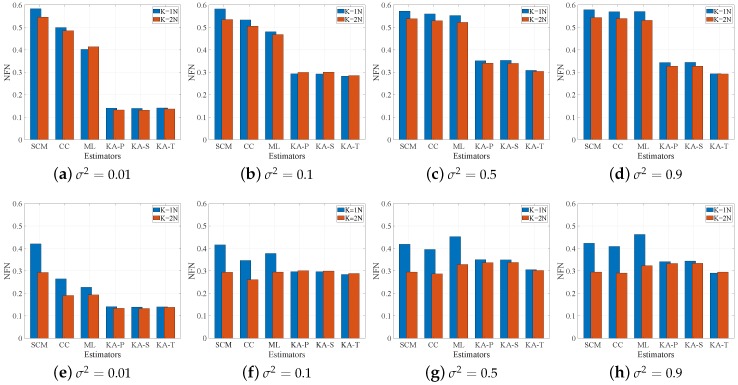
NFN under different values of the perturbation level (**a**–**d**) in compound-Gaussian clutter; (**e**–**h**) in Gaussian clutter.

We calculate the mean of 1000 Monte Carlo experiments of the NFN to obtain the NFN estimation values of each estimator, shown in Figure 2. From the estimation error results in the compound-Gaussian clutter (Figure 2a–d), we find that the SCM estimator suffers from significant performance loss, and hence the CC and ML estimators using the SCM as the basic estimator have serious estimation error, though the $\sigma^{2}$ is small. For the proposed estimators, in the compound-Gaussian clutter, the KA-T estimator has the smallest estimation error, which suggests that the KA-T has the best detection performance among the proposed estimator. Compared with the SCM, CC and ML estimators, the proposed estimators have better estimation accuracy than them in the compound-Gaussian clutter. In the Gaussian clutter (Figure 2e–h), the estimators have similar estimation accuracy when K=2N. When K=N, the proposed estimators have better estimate results than the SCM, CC and ML estimators.

In general, from Figure 2, when in the Gaussian clutter and the number of secondary data are sufficient, all three estimators have similar estimation performance. In other cases, the proposed estimators are better than the SCM, CC and ML estimators. Moreover, among proposed estimators, the KA-T estimator provides the best estimate.

### 4.2. Detection Performance

To further illustrate the effectiveness of our estimators, we address the problem of detecting a known complex signal vector s in received data vector, which can be formulated as a binary hypotheses test

(25) $$\left\{\begin{array}{cccc} H_{0}: & y=r_{0}, & y_{k}=r_{k}, & k=1...K\\ H_{1}: & y=as+r_{0}, & y_{k}=r_{k}, & k=1...K \end{array}\right.$$

where $s=\frac{1}{\sqrt{N}}{\left[1,e^{\left(j2\pi f_{d}\right)},\cdots e^{\left(j2\pi \left(N-1\right)\right)f_{d}})\right]}^{T}$, *a* is an unknown deterministic parameter, which accounts for both the target and the channel effects. $f_{d}$ is the normalized frequency of the target and set to be $f_{d}=0.1$.

Then, we use the ANMF detector to analyse the detection performance of our proposed covariance estimators. The structure of ANMF detector is defined as [51,52]

(26) $$\frac{{\left|s^{H}{\hat{R}}^{-1}y\right|}^{2}}{\left(s^{H}{\hat{R}}^{-1}s\right)\left(y^{H}{\hat{R}}^{-1}y\right)}\underset{H_{0}}{\overset{H_{1}}{≷}}\;\eta_{\mathrm{ANMF}}$$

where $\eta_{\mathrm{ANMF}}$ is the threshold of ANMF, $\hat{R}$ can be replaced by ${\hat{R}}_{\mathrm{KA}-P}$, ${\hat{R}}_{\mathrm{KA}-S}$, ${\hat{R}}_{\mathrm{KA}-T}$, ${\hat{R}}_{\mathrm{SCM}}$, ${\hat{R}}_{\mathrm{CC}}$ and ${\hat{R}}_{\mathrm{ML}}$.

The standard Monte Carlo technique based on 100/PFA independent trails is utilized to obtain the decision thresholds for a given probability of false alarm PFA. In this paper, $\mathrm{PFA}=10^{-3}$ to alleviate the computational burden. The detection probabilities (PD) are computed via 100,000 Monte Carlo experiments. In the compound-Gaussian case, the signal-to-clutter ratio (SCR) is defined as [47]

(27) $$\mathrm{SCR}=\frac{E\{a^{2}\}}{E\{\tau \}}$$

where $E\{\tau \}=\frac{\mu }{\left(\lambda -1\right)}$ for λ>1. In the Gaussian case, we set τ=1.

**Figure 3:**
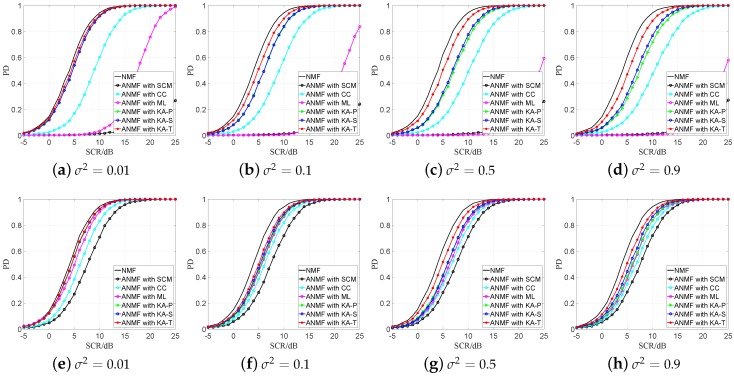
PD versa SCR with different values of the perturbation level in compound-Gaussian clutter PFA = 10^−3^, (**a**–**d**) *K* = 1*N*; (**e**–**h**) *K* = 2*N*.

**Figure 4:**
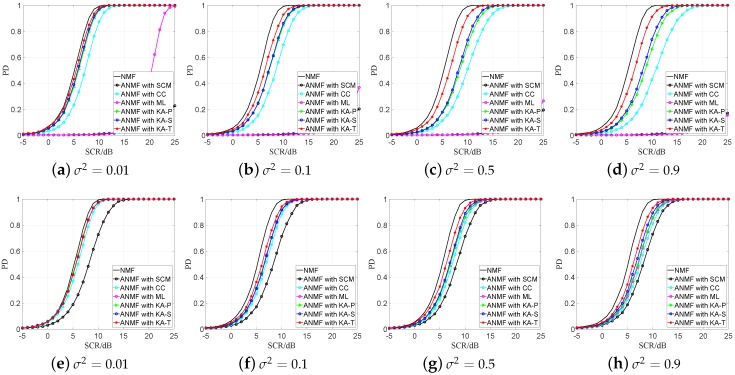
PD versa SCR with different values of the perturbation level in Gaussian clutter PFA = 10^−3^, (**a**–**d**) *K* = 1*N*; (**e**–**h**) *K* = 2*N*.

The sensor detection results are shown as Figure 3 and Figure 4 in the compound-Gaussian and Gaussian environments, respectively. It is shown that the detection performance of the proposed estimators are better than that of the other three estimators in most cases. In the compound-Gaussian case (Figure 3), compared with the ML estimator, the ANMF detector with proposed estimators have a significant advantage when secondary data are insufficient. When K=2N and $\sigma^{2}\geq 0.1$, the detection performance of the ML estimator is close to the KA-P and KA-S estimators. For the CC estimator, it is more robust than the ML estimator when K=N. But compared with proposed estimators, it still suffers much detection loss because of the non-Gaussian clutter. When prior knowledge is accurate, e.g., $\sigma^{2}=0.01$, the three proposed estimators have nearly the same detection performance. When the prior knowledge becomes coarse, the detection performance of proposed estimators begin to differentiate. The best is the KA-T estimator, followed by the KA-S and KA-P estimator.

The sensor detection results in the Gaussian clutter case is shown in Figure 4. The proposed estimators have better performance when the secondary data are insufficient because the proposed estimators can use the prior knowledge more effectively which can be found in Figure 1c. When the secondary data are sufficient, all the KA estimators have similar detection performance, but the KA-T estimator still has a small advantage.

From Figure 3 and Figure 4, we find that the proposed estimators have better detection performance than the SCM, CC and ML estimators, especially under the sample starvation situation. The ML and SCM estimators are sensitive to the number of secondary data, while the CC estimator has relatively robust property in secondary data changing. Among the proposed estimators, the KA-T has the best detection performance, which is similar to the result in Figure 1 and Figure 2.

### 4.3. IPIX Radar Sensor System Data

In this section, we use the ANMF detector with the KA-P, KA-S, KA-T, SCM, CC and ML estimators with one set of real data collected by the McMaster IPIX radar sensor system. The IPIX radar sensor system is a transportable experimental radar system designed and constructed at McMaster University.

A detailed statistical analysis of the adopted real data has been conducted in [38,54,55,56]. We use the *19980223_170435_ANTSTEP.CDF* data, which consists of 60,000 coherent pulse trains and 34 range cells, to analyze the proposed detector. The sensor system measurements were collected in winter 1998 using the McMaster IPIX radar in Grimsby, on the shore of Lake Ontario, between Toronto and Niagara Falls. This sensor system is a fully coherent X-band radar, with advanced features such as dual transmit/receive polarization, frequency agility, and stare/surveillance mode. Moreover, it has been upgraded since the measurement of the Dartmouth data in 1993. More specifically, the dynamic range has been improved from 8 bits to 10 bits, so that strong target and weak clutter signals can be observed simultaneously without clipping or large quantization error. The sensor system parmeters are shown in Table 2.

**Figure 5:**
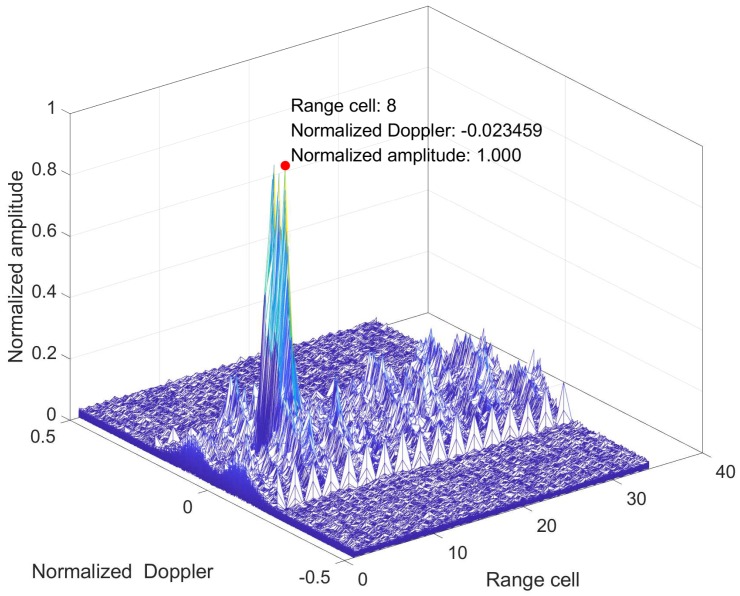
Range–Doppler image of IPIX clutter data.

The Range–Doppler image is shown as Figure 5.

It is shown that the strongest clutter appear at the 8th range cell. Therefore, we choose the 8th range cell as the CUT, exploiting K=N,2N surrounding cells $\left(\left[1,7\right]⋃\left[9,19\right]\right)$ as training data. Moreover, because of the normalized Doppler of clutter is near zero and radar transmits the coherent pulses, the covariance meets the prior condition of symmetry, persymmetry and Toeplitz structure. We apply the statistical tests for all the available N=8 temporal samples, the total number of different windows is 60,000−N+1. According to [53], we use the MLE to estimate λ=2.4072 and μ=1.36.

**Figure 6:**
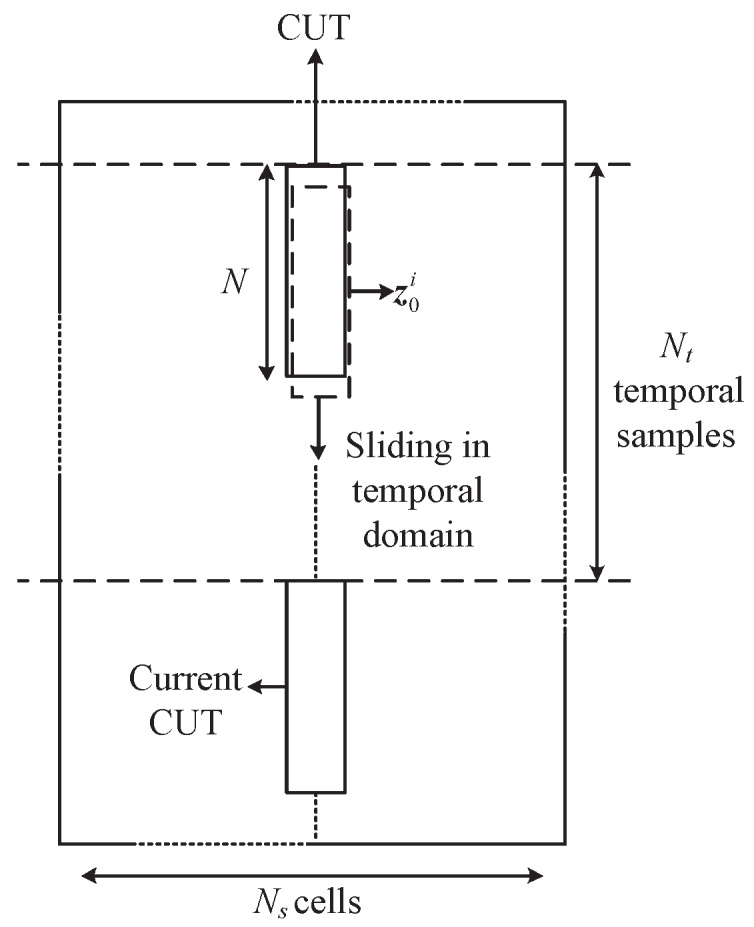
Procedure for estimating $R_{A}$.

For the prior covariance, the SAR imagery [35] and digital elevation model (DEM) [37] are not always getatable, physics-based models [36] may have model mismatch. Compared with above prior knowledge, the historical environment data are more accessible and timely. Therefore, according to [33,57], we use the previous $N{}_{t}=107$ temporal samples of current CUT as the historical measure data to estimate 100 covariance matrices. Then, we obtain the prior knowledge covariance matrix $R_{A}$ of the CUT via calculating the mean of the 100 covariance matrices. As shown in Figure 6, we slide the window to obtain the *i*th covariance matrix estimation ${\hat{R}}_{0}^{i}$, namely

(28) $${\hat{R}}_{0}^{i}=z_{0}^{i}z{{}_{0}^{i}}^{H}$$

where $z_{0}^{i}$ is the *i*th sliding normalized vector in the CUT. Then, we average all of $R_{0}^{i}$ to obtain the prior knowledge covariance matrix

(29) $$R_{A}=\frac{1}{N{}_{t}-N+1}\sum_{i=1}^{N{}_{t}-N+1}{\hat{R}}_{0}^{i}$$

After the above process, we add a point target in the 8th range cell for different SCRs and set the normalized Doppler of the target as $f_{d}=0.1$. We use the first 50,000 pulses via standard Monte Carlo technique to obtain the detection thresholds for a given probability of false alarm ($\mathrm{PFA}=10^{-3}$) and use the remaining 10,000 pulses to obtain the PD of each detector.

**Figure 7:**
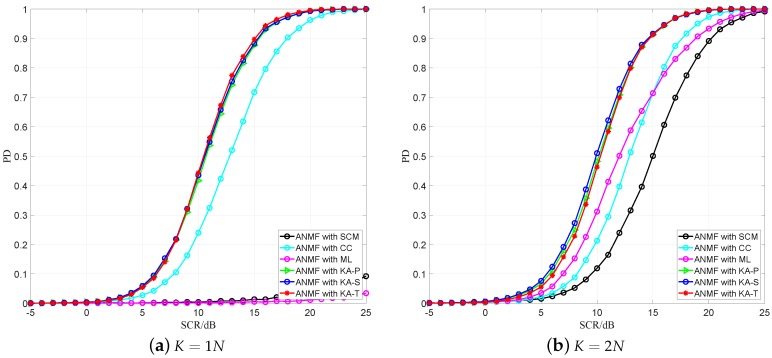
PD versa SCR for IPIX Radar data under different number of secondary data.

The sensor detection results are shown in Figure 7. Like the simulation results, the ANMF detector with the ML estimator is sensitive to the number of secondary data. When K=N the ANMF detector with the ML estimator and the ANMF detector with the SCM estimator are fail to detect the target. While, the proposed estimators and the CC estimator work well when secondary data are insufficient and the proposed estimators can obtain a better detection performance. For the proposed estimators, when K=N, the KA-T estimator has a little advantage, and when K=2N, the proposed KA-S estimators have similar performance.

According to the simulation results and IPIX radar sensor system data, we can summarize that the KA-T estimator has the best performance both in estimation and detection under the choosen condition, followed by the KA-S and KA-P estimator. Compared with the existing KA methods, i.e., the CC and ML estimator, the proposed estimators can perform better, especially in the compound-Gaussian clutter.

In addition, according to the Section 3, each structure has special application scene and some papers [58,59] have proposed methods to recognize the structure property of the covariance to help using the structure information.

## 5. Conclusions and Future Work

In this paper, we have considered the problem of covariance estimation with insufficient secondary data in compound-Gaussian clutter. We have proposed three knowledge-aided covariance estimators and applied the estimators to radar sensor signal detection.

According to the experimental results using simulated and measured sensor data, the KA-T estimator has the best performance both in estimation and detection, followed by the KA-S and KA-P estimator. Compared with the existing KA methods, i.e., the CC and ML estimators, the proposed estimators can perform better, especially with compound-Gaussian clutter.

Possible future research tracks might concern the design of the knowledge-aided covariance estimator based on other geometric means, such as Log–Euclidean mean, Root–Euclidean mean, Powe–Euclidean mean, and Cholesky mean.

## Figures and Tables

**Table 1:** Three knowledge-aided structured covariance estimators.

Estimator	α
${\hat{R}}_{\mathrm{KA}-P}=\frac{1}{K}\sum_{i=1}^{K}\left({\hat{\alpha }}_{P}R_{A}+\left(1-{\hat{\alpha }}_{P}\right){\hat{R}}_{kP}\right)$	${\hat{\alpha }}_{P}=\frac{\sum_{k=1}^{K}{∥{\hat{R}}_{kP}-{\hat{R}}_{P}∥}_{F}^{2}}{\sum_{k=1}^{K}{∥R_{A}-{\hat{R}}_{kP}∥}_{F}^{2}}$
${\hat{R}}_{\mathrm{KA}-S}=\frac{1}{K}\sum_{i=1}^{K}\left({\hat{\alpha }}_{S}R_{A}+\left(1-{\hat{\alpha }}_{S}\right){\hat{R}}_{kS}\right)$	${\hat{\alpha }}_{S}=\frac{\sum_{k=1}^{K}{∥{\hat{R}}_{kS}-{\hat{R}}_{S}∥}_{F}^{2}}{\sum_{k=1}^{K}{∥R_{A}-{\hat{R}}_{kS}∥}_{F}^{2}}$
${\hat{R}}_{\mathrm{KA}-T}=\frac{1}{K}\sum_{i=1}^{K}\left({\hat{\alpha }}_{T}R_{A}+\left(1-{\hat{\alpha }}_{T}\right){\hat{R}}_{kT}\right)$	${\hat{\alpha }}_{T}=\frac{\sum_{k=1}^{K}{∥{\hat{R}}_{kT}-{\hat{R}}_{T}∥}_{F}^{2}}{\sum_{k=1}^{K}{∥R_{A}-{\hat{R}}_{kT}∥}_{F}^{2}}$

**Table 2:** The system parameters of the IPIX radar sensor.

19980223_170435_ANTSTEP.CDF	
Date and time (UTC)	1998/02/23 17:04:35
RF frequency	9.39 GHz
Pulse length	100 ns
Pulse repetition frequency	1000 Hz
Radar azimuth angle	$346.75^{\circ }$
Range	3500–4000 m
Range resolution	15 m
Radar beamwidth	$0.9^{\circ }$

## Appendix A

The detail formula derivation of $\bar{R}$ is obtained in this Appendix. Equation (9) be recasted as.

(A1) R¯=1KR−αRA−1−αR^kF2=1K∑k=1Kα2RA−R^kF2+1K∑k=1KR−R^kF2−1K∑k=1K2αRetrRARH−RAR^kH−R^kRH+R^kR^kH=1K∑k=1Kα2RA−R^kF2−1K∑k=1K2αReR^k−RF2+1K∑k=1KR−R^kF2−1K∑k=1K2αRetrRARH−RAR^kH+RR^kH−RRH

According to (A1), we obtain

(A2) R¯=α2K∑k=1KRA−R^kF2−1K∑k=1K2αReR^k−RF2+c−2αK∑k=1KRetrRARH−RAR^kH+RR^kH−RRH

Then we take derivative of (A2) with respect to α and nulling the result we obtain

(A3) α^=∑k=1KR^k−RF2∑k=1KRA−R^kF2+∑k=1KRetrR0RH−R0R^kH+RR^kH−RRH∑k=1KRA−R^kF2

It is noted that R is unknown, but according to [23] R can be replaced by $\hat{R}$ in practice, namely

(A4) $$R\approx \hat{R}=\frac{1}{K}\sum_{k=1}^{K}{\hat{R}}_{k}$$

Taking $\hat{R}$ into (A3) then the second term of (A3) is equal 0. Therefore we can obtain the final result given in (11).
